# Case Report: Brentuximab Vedotin Associated Acute Pancreatitis in a Pediatric Hodgkin Lymphoma Patient: Case Report and Literature Review

**DOI:** 10.3389/pore.2022.1610445

**Published:** 2022-08-12

**Authors:** Ewelina Truszkowska, Marta Andrzejewska, Cyntia Szymańska, Agnieszka Wziątek, Katarzyna Derwich

**Affiliations:** ^1^ Faculty of Medicine, Poznan University of Medical Sciences, Poznan, Poland; ^2^ Department of Pediatric Oncology, Hematology and Transplantology, Institute of Pediatrics, Poznan University of Medical Sciences, Poznan, Poland

**Keywords:** Hodgkin lymphoma, pediatric oncology, brentuximab vedotin, acute pancreatitis, side effects

## Abstract

Brentuximab vedotin is a conjugate drug used mainly in Hodgkin lymphoma, systemic and primary cutaneous anaplastic large cell lymphomas, and CD30-expressing peripheral T-cell lymphoma. We report a unique case of acute pancreatitis associated with brentuximab vedotin in a 17-year-old male patient suffering from classical Hodgkin lymphoma. Diagnosed in 2020, the patient was classified to an intermediate therapeutic group and disease’s grade was IIIAE. The patient was treated with brentuximab vedotin and bendamustine in the third line. Two weeks after the drug administration, the patient developed acute epigastric pain. Laboratory and radiological findings confirmed the clinical suspicion of acute pancreatitis that was managed with opioid pain medications, meropenem, parenteral nutrition, ondansetron and omeprazole. This is the first case report of brentuximab vedotin-associated acute pancreatitis in the pediatric patient reported in the literature to the best of our knowledge.

## Introduction

Hodgkin lymphoma (HL) is one of the most common neoplasms among adolescents [1]. Thanks to advances in its treatment, a 5-year event-free survival rate exceeds 90% [2]. Adverse outcomes in the remaining 10% of patients are related to various factors, including advanced stage of disease, bulky disease, pleural and cardiac effusion [3–6]. In addition, male sex and initially slow response to commencing cycles of chemotherapy are adverse prognostic factors [3, 7]. Global results of HL treatment are excellent, yet refractory HL remains a therapeutic challenge. Due to the small percentage of complex cases, a limited number of clinical trials focus on refractory Hodgkin disease [2]. Moreover, another aspect that should be considered is the questionable ethics of clinical trials conducted among pediatric patients. Some of the agents may have different pharmacokinetics and side effects that may even affect a child’s development, hence reluctance to conduct such trials [8].

In the pediatric population, the leading protocol used for the first-line treatment of Hodgkin lymphoma was established by the European Network-Paediatric Hodgkin Lymphoma Study Group (EuroNet-PHL) [2]. This trial’s strategy focuses on the restriction of radiation therapy by including patients with Deauville scores 4 and 5 only and augmentation of chemotherapy intensity in intermediate and advanced HL [2]. Second-line and further lines of treatment comprise various schemes of chemotherapy, which may be combined with brentuximab vedotin (BV), nivolumab, or followed by autologous hematopoietic cell transplantation [2].

Brentuximab vedotin is an anti-CD30 directed antibody-drug conjugate to auristatin, initially used in adult patients with HL and anaplastic large cell lymphoma, refractory and relapsed [9, 10]. Now, its use is investigated among the pediatric population in several clinical trials [11, 12]. The pivotal study conducted by Locatelli et al. showed BV is an agent able to reduce late toxicity and adverse events among pediatric patients that stems from the administration of traditional chemotherapy [12]. In addition, it proved to help lead to autologous stem cell transplantation in these patients. The most common adverse reactions include peripheral neuropathy and neutropenia [11, 12]. While acute pancreatitis has been described in a few case series and case reports, all previously described patients were adults [13–15]. Brentuximab vedotin is the first agent that should be considered while planning the treatment of R/R HL with any novel agents [12]. The combination of brentuximab vedotin and bendamustine was introduced after studies conducted by Vinti et al. and McMillan et al. showed promising data for pediatric patients with little data on severe toxicity [16, 17].

In this case report, we describe a 17-year-old male patient with refractory Hodgkin lymphoma that was administered brentuximab vedotin and bendamustine (B) in his 4th cycle of treatment. He developed acute pancreatitis 2 weeks after the first infusion of BV. We also review other BV-associated pancreatitis case reports described in the literature, elucidate the possible mechanism of this adverse event and present the consensus on the treatment of AP in pediatric oncological patients.

## Case Report

A currently 17-year-old overweight male patient (1.79 m, 90 kg, BMI 28.09) with a bulky (>200 ml) mediastinal tumor was diagnosed with nodular sclerosis classical HL, IIIAE grade, intermediate therapeutic group (TL-2) at the age of 16. Due to life-threatening pressure of the mass and superior vena cava syndrome, as well as venous thrombosis, the debulking operation was performed. At first, steroid treatment was implemented. Later, the patient was enrolled in the EuroNET-PHL-C2 clinical trial [18]. The patient was administered OEPA (vincristine sulfate 1.5 mg/m^2^, etoposide phosphate 125 mg/m^2^, doxorubicin hydrochloride 40 mg/m^2^, prednisone 60 mg/m^2^) chemotherapy. After two cycles, his group was changed to advanced (TL-3) due to mass penetration into the spinal canal in the thoracic Th2/Th3 segment, and he was qualified to COPDAC (cyclophosphamide 600 mg/m^2^, vincristine sulfate 1.4 mg/m^2^, dacarbazine 250 mg/m^2^, prednisone 40 mg/m^2^). Despite intensified chemotherapy regimen, an interim PET-CT scan showed progression (Deauville 5). Therefore, treatment was changed to IGEV (ifosfamide 2000 mg/m^2^, gemcitabine 800 mg/m^2^, vinorelbine 20 mg/m^2^, prednisolone 100 mg/m^2^). Later, brentuximab vedotin and bendamustine were introduced: 188.1 mg (90 mg/m^2^) bendamustine was administered twice and there was one infusion of 162 mg (1.8 mg/kg) brentuximab vedotin.

**FIGURE 1:**
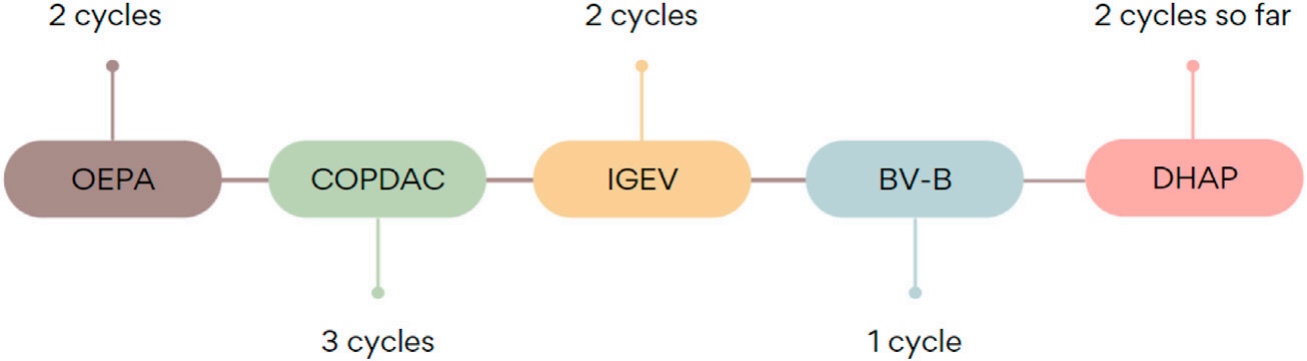
The sequence and number of chemotherapy regimens administered.

After 2 weeks from the first brentuximab vedotin administration, the patient presented to the emergency department with abdominal and back pain and vomiting. Physical examination revealed tachycardia, abdominal bloatedness, and disseminated pain in palpation of the whole abdomen. In laboratory tests inflammatory reaction was observed (CRP 5.32 mg%, leucocytes 17 990/mm^3^, neutrophils 15 870/mm^3^) and levels of pancreatic enzymes were increased (amylase 1665 U/L, lipase 3853 U/L). Triglycerides levels were within reference values—118 mg/dl and rose up to maximum of 170 mg/dl on the 10th day of hospitalization. Ultrasound investigations revealed a slightly enlarged pancreas as compared to age (head 1.9 cm, body 2.6 cm, tail 2.4 cm), peripancreatic adipose tissue of increased echogenicity and sign of free fluid in the peritoneal cavity. CT scan confirmed the inflammatory reaction of tissues surrounding the pancreas. The patient was administered pain medications - metamizole and tramadol, fluid therapy, meropenem, ondansetron and omeprazole. A gastric tube was also used. Due to the increasing severity of pain, its treatment was changed to morphine and nalbuphine. Parenteral feeding was introduced. During the following days, there was a significant increase in inflammatory parameters (maximum C-reactive protein 42 mg%), and therefore, the antibiotic therapy was intensified. Gradual clinical improvement of the patient and normalization of pancreatic enzymes was observed. However, the patient complained of strengthening symptoms resulting from the underlying Hodgkin lymphoma. The increase of inflammatory markers was also observed. Chest CT confirmed the progression of the disease and the rise in tumors’ infiltration. Oral steroid therapy was introduced and DHAP (cytarabine 2000 mg/m^2^, cisplatin 100 mg/m^2^, dexamethasone 40 mg/m^2^) chemotherapy regimen was planned. It was followed without any severe side-effects, except transient increase of transaminase level. The patient is still being treated with a DHAP regimen with a non-satisfactory result. It is planned he will be given nivolumab as the last-chance treatment and followed by autologous hematopoietic stem cell transplantation. Illustration of chemotherapy sequence is shown in Figure 1.

## Discussion

Despite an excellent overall prognosis, refractory HL still poses a therapeutic challenge. The optimal therapy in R/R Hodgkin disease in pediatric patients has not been established due to the scarcity of data and lack of randomized trials that could compare possible approaches and their effectiveness. It is unknown whether standard-dose chemotherapy is better than high dose chemotherapy followed by autologous stem cell transplant; only small cohort national trials show that high dose chemotherapy is usually not needed [2]. EuroNet Pediatric Hodgkin Lymphoma group has thus issued guidelines on approaching refractory HL considering new possible therapies that include brentuximab vedotin. The panel also considers the stratification of patients into three subgroups depending on the time of relapse and previous treatment. The described patient qualifies to the high-risk group that includes patients that failed to achieve remission in PET-CT after two lines of salvage standard-dose therapy. Such patients are candidates for treatment with conventional high dose chemotherapy, autologous stem cell transplantation and additional therapies, i.e., biological drugs or experimental strategies.

The use of BV-B (brentuximab vedotin and bendamustine) has proved to have a non-dangerous safety profile. The use of these agents provokes a long-lasting response to the treatment and is a bridge to autologous stem cell transplantation in the future, providing the possibility of stem cell mobilization [2].

It is worth mentioning that the patient is overweight (BMI 28.09) and the dose of brentuximab vedotin is weight-dependent. The required dosage of BV for patients from 12 years old weighing between 50 and 100 kg is 1.8 mg/kg every 3 weeks or 1.2 mg/kg every 2 weeks. However, in younger children [19], treatment results were worse using these doses. Suri et al. elucidated in their pharmacokinetics analysis that the recommended dose suitable for all the patients (up to 100 kg) is 71.5 mg/m^2^ every 3 weeks and 47.7 mg/m^2^ every 2 weeks and it provides the same concentrations of the drug (AUC, area under curve). In the case of our patient, the calculated dose would therefore be 148.7 mg, which is a slightly smaller dose than the one calculated based on the patient’s weight. Nevertheless, statistical correlation between the dose of BV and acute pancreatitis or different adverse events has not been proven in literature yet. In the case of our patient, it is essential to keep in mind that high body mass index and adiposity increase the risk of AP and being overweight is a negative prognostic factor of AP severity [20–23].

## Literature Review

### Other BV-Induced Pancreatitis Cases Described in the Literature

Almost one-fifth of the BV patients present abdominal pain, which raises the question of whether BV-induced pancreatitis is underdiagnosed or truly rare. So far, only a few cases of brentuximab vedotin induced pancreatitis in patients with HL have been reported in the literature, of which two were fatal, but none of them occurred in the pediatric population. One fatal pancreatitis was reported at the beginning of the clinical trial (NCT01476410) [24], which led to the exclusion of patients with a history of AP. Then one patient without such history developed this toxicity. Urru et al. reported the first known case of BV-induced pancreatitis in a Hodgkin lymphoma patient [14]. Even though the patient developed symptoms of acute pancreatitis a few days after the second infusion of the drug, which differs, symptoms and clinical outcome were similar to our case and following case reports. Gandhi et al. describe a case series of 8 patients with acute pancreatitis that emerged in a median time of 26 days after the first exposure to brentuximab vedotin and 12 days after its recent administration [15]. Maradana et al. reported severe hypertriglyceridemia-induced acute pancreatitis 2 weeks after the first brentuximab treatment in a patient with T-cell skin lymphoma [13]. As mentioned earlier, BV is also useful in the treatment of CD30^+^ ALCL [12]. Chen et al. describe the first case of fatal pancreatitis on the 13th day in a patient suffering from ALCL [25]. All the reported cases of BV-induced pancreatitis are listed in Table 1. Interestingly, there is a case of polatuzumab-vedotin-piiq-induced pancreatitis reported in literature. No previous pancreas-related adverse events were reported during clinical trials with this agent [26].

**TABLE 1:** The summary of all reported cases of pancreatitis associated with brentuximab vedotin treatment.

	Sex of the patient	Age	Disease	Cycle	Outcome	References
1	F	65	HL	2	Grade 5, death	[24]
2	F	34	HL	1	5	[15]
3	M	23	HL	2, 5	4	
4	M	52	HL	2	3	
5	F	25	HL	3	3	
6	F	63	ALCL	1	3	
7	M	51	HL	1	3	
8	M	38	HL	1	3	
9	M	32	ALCL	1	Grade 5, death	[25]
10	M	38	cutaneous gamma/delta T-cell lymphoma	1	Good; continuation of BV treatment	[13]
11	M	65	HL	2	Grade 3, continuation of BV treatment	[14]
12	M	17	HL	1	Good outcome, change of regimen	This case report

The vast majority of BV-induced AP cases are revealed after the first and second administration of the agent. In our case, the patient developed acute pancreatitis after 2 weeks from first and only brentuximab vedotin intake, which is consistent with previous reports. Three patients continued BV intake, 2 of which did without any adverse events and one of the patients developed pancreatitis again after three other infusions. This data is too scarce to say whether the administration of BV should be discontinued in all the cases or can be resumed with a benefit for the patient. For now, its readministration should be discouraged. Our patient had no chance to profit from this treatment as one cycle of BV proves too little to objectively assess its impact.

### Patomechanism of BV-Induced Pancreatitis

The cause of BV-induced pancreatitis is still uncertain. Nevertheless, another case report suggests targeting variable low-level CD30 in the pancreas due to higher expression of in patients than in mouse immunoglobulin controls [15]. Also, other considered mechanisms of this adverse effect are circulating unconjugated monomethylauristatin E or the occurrence of isolated CD30-positive malignant cells in the pancreas [15]. Host-dependent reasons are also considered, such as predisposing genetic variants, which the best described are *PRSS1, CTRC, SPINK1, CFTR* [27]*.*

### Acute Pancreatitis as a Side Effect of Drugs Used in Pediatric Oncology

Acute pancreatitis may be caused by various drugs, also those used in pediatric oncology. However, diagnosis of drug-induced AP is often unclear and based only on clinical suspicion rather than evidence. In addition, many case reports do not exclude more common reasons for AP, such as gallstones, alcohol, hypertriglyceridemia, which makes it a real challenge to find the actual frequency of drug-associated pancreatitis [28].

In pediatric oncology, steroids, antibiotics such as tetracyclines and trimethoprim/sulfamethoxazole, asparaginase and mercaptopurine are associated with drug-induced AP [29]. The most widely known drug to cause AP is asparaginase, with the first case reported in the 1970s and one of the most important reasons to stop this drug [29, 30]. The evolution of asparaginase treatment and its new generations was crucial in improving the treatment results [31]. Approximately 2–18% of patients administered with asparaginase are affected by that side reaction [31]. Pathophysiology is yet unknown but is probably related to the mechanism of action of this drug. Asparaginase depletes external sources of asparagine, which is detrimental for lymphoblasts and affects organs with high protein expenditure like the pancreas [30]. The risk of AP is independent from dose or route of administration [30]. Another critical factor is the concomitant use of steroids, especially dexamethasone [32].

Possibly important risk factors are age, concurrent therapy with other anticancer drugs, some genetic variants and hypertriglyceridemia [30]. Asparaginase therapy may be reintroduced only when amylase or lipase is mildly elevated and the patient presents no clinical symptoms [30]. Nevertheless, more than 60% of re-exposed patients may have a relapse [33]. Compared to brentuximab vedotin, clinical symptoms, diagnostics methods and general outcomes are similar to those in generally healthy children [29]. However, immunodeficiency should be kept in mind and the adverse effects of other oncological drugs.

Bortezomib is another drug used in reinduction therapy of ALL in children that have been described to provoke acute pancreatitis [34]. Its mechanism of action is based on a reversible inhibition of proteasome subunit 26s, thereby inhibiting its chymotrypsin activity. However, this side effect is rare, occurring in 0,1–2% of cases [35]. The mechanism of inflammation is still unknown, but direct drug toxicity or allergic and immunological causes are considered [35]. Elevation of pancreatic enzymes is observed chiefly a few days after drug administration. It is possible to continue bortezomib therapy [35].

AP should also be considered an adverse effect of mercaptopurine; however, its frequency depends on the treated disease. For example, in inflammatory bowel disease patients, mercaptopurine-associated AP is a well-described complication of treatment, occurring in 2–6% of cases [36]. When it comes to hematological diseases, fewer reports can be found. Nevertheless, there are described cases in acute lymphoblastic leukemia [37].

Also, cytarabine may induce AP. Very few papers report that side effect in acute myeloblastic leukemia [38]. Therefore, the mechanism is not found, but previous exposure to asparaginase is considered.

AP may also be concerning in the stem cell transplantation scenario as it may be caused by tacrolimus or cyclosporine [39]. Very few case reports can be found with tacrolimus-associated AP, most of them after solid organ transplantation [40]. The majority of them reported high or very high concentrations of tacrolimus and cyclosporine was implemented instead [40].

### Treatment of Pancreatitis in Pediatric Oncological Patients

Management of AP in leukemia/lymphoma patients should be more intensive than in less complicated cases. Due to the poorer oncological outcome after canceling the drug, it is imperative to assure whether there is pancreatitis, its severity and therefore a need to stop the treatment. In an asymptomatic course, when only enzymes are elevated (chemical pancreatitis), it is possible to continue drug administration [41].

Pharmacotherapy of AP remains a controversial topic and the use of antibiotics, protease inhibitors and octreotide are widely discussed. Prophylactic use of antibiotics is generally discouraged until infected necrosis is proved [42]. However, even in the case of acute necrotizing pancreatitis, the data is inconsistent. Two controlled, double‐blind studies and one meta-analysis show no benefit of antibiotics therapy, while two separate meta‐analyses concluded that the patients with proven pancreatic necrosis should receive either imipenem or meropenem prophylaxis [29, 43, 44, 45].

On the other hand, when l-asparaginase induced AP is concerned, authors suggest using broad-spectrum antibiotics, especially imipenem or meropenem [46]. This approach is seen in adult ALL patients as well, with recognition of no clear evidence that antibiotics prevent the development of sepsis or other complications [47].

All authors agree that supportive care is crucial [29]. Fluid therapy, proper nutrition and pain management are undoubtedly critical. Patient monitoring (cardiovascular, pulmonary and renal parameters) is essential to determine AP complications quickly.

### Other Rare Complications and Toxicities Associated With Brentuximab Vedotin

BV may cause many more or less serious side effects, apart from AP, such as peripheral neuropathy, nausea, fatigue, neutropenia, diarrhea, pyrexia, vomiting, arthralgia, pruritus, myalgia [48].

The most fatal and rare toxicity is progressive multifocal leukoencephalopathy (PML) caused by JC infection [49]. To the best of our knowledge, nine cases have been reported so far and at least six of them are lethal [49]. PML is often a fatal disease caused by the reactivation of JCV. Currently, it is associated with HIV infection, yet less known is the fact that before the spread of HIV, patients with lymphoproliferative disorders were ones who mostly suffered from PML [49]. The disease is associated with chemotherapy, immunosuppressive medications as well as monoclonal antibodies, including natalizumab, rituximab and brentuximab vedotin [49]. The most common symptoms of PML are motor weakness, gait abnormality, sensory loss, speech disorders and visual abnormalities [50]. Product monograph of brentuximab vedotin accentuates the importance of symptom monitoring and proper diagnostics as soon as they emerge [51]. Drugs should be stopped in PML patients and are contraindicated for patients who have had PML [51].

## Conclusion

It should be kept in mind that any epigastric pain in a patient that intakes brentuximab vedotin should be closely diagnosed as it may be a symptom of acute pancreatitis. While this drug is characterized by an overall safe profile and proves to be very efficient in treating refractory Hodgkin disease, acute pancreatitis is a severe adverse reaction, which requires immediate, adequate treatment and may lead to further complications. The direct link between BV and acute pancreatitis cannot be proved. Nonetheless, based on previous case reports, we wish to highlight such possibility and report such association in the pediatric patient.

## Data Availability

The original contributions presented in the study are included in the article/supplementary material, further inquiries can be directed to the corresponding author.

## References

[1] Miller KD Fidler-Benaoudia M Keegan TH Hipp HS Jemal A Siegel RL Cancer Statistics for Adolescents and Young Adults, 2020. CA Cancer J Clin (2020) 443:6–59. 10.3322/caac.21637 32940362

[2] Daw S Hasenclever D Mascarin M Fernández-Teijeiro A Balwierz W Beishuizen A Risk and Response Adapted Treatment Guidelines for Managing First Relapsed and Refractory Classical Hodgkin Lymphoma in Children and Young People. Recommendations from the EuroNet Pediatric Hodgkin Lymphoma Group. HemaSphere (2020) 4:e329. 10.1097/HS9.0000000000000329 32072145 PMC7000476

[3] Smith RS Chen Q Hudson MM Link MP Kun M Weinstein H Prognostic Factors for Children with Hodgkin's Disease Treated with Combined-Modality Therapy. J Clin Oncol (2003) 21:2026–33. 10.1200/JCO.2003.07.124 12743158

[4] Schwartz CL Chen L McCarten K Wolden S Constine LS Hutchison RE Childhood Hodgkin International Prognostic Score (CHIPS) Predicts Event-free Survival in Hodgkin Lymphoma: A Report from the Children's Oncology Group. Pediatr Blood Cancer (2017) 64(4):e26278. 10.1002/pbc.2627810.1002/pbc.26278 PMC5702912 27786406

[5] McCarten KM Metzger ML Drachtman RA Pei Q Friedman DL Schwartz CL Significance of Pleural Effusion at Diagnosis in Pediatric Hodgkin Lymphoma: A Report from Children's Oncology Group Protocol AHOD0031. Pediatr Radiol (2018) 48(12):1736–44. 10.1007/s00247-018-4197-6 30014200 PMC6208959

[6] Marks LJ McCarten KM Pei Q Friedman DL Schwartz CL Kelly KM Pericardial Effusion in Hodgkin Lymphoma: A Report from the Children’s Oncology Group AHOD0031 Protocol. Blood (2018) 132(11):1208–11. 10.1182/blood-2018-02-834465 30061157 PMC6137557

[7] Hutchings M Loft A Hansen M Pedersen LM Buhl T Jurlander J FDG-PET after Two Cycles of Chemotherapy Predicts Treatment Failure and Progression-free Survival in Hodgkin Lymphoma. Blood (2006) 107(1):52–9. 10.1182/blood-2005-06-2252 16150944

[8] Joseph PD Craig JC Caldwell PH. Clinical Trials in Children. Br J Clin Pharmacol (2015) 79:357–69. 10.1111/bcp.12305 24325152 PMC4345947

[9] Bazarbachi A Boumendil A Finel H Mohty M Castagna L Blaise D Brentuximab Vedotin for Recurrent Hodgkin Lymphoma after Allogeneic Hematopoietic Stem Cell Transplantation: A Report from the EBMT Lymphoma Working Party. Cancer (2019) 125:90–8. 10.1002/cncr.31755 30351488

[10] O'Connor OA Lue JK Sawas A Amengual JE Deng C Kalac M Brentuximab Vedotin Plus Bendamustine in Relapsed or Refractory Hodgkin's Lymphoma: an International, Multicentre, Single-Arm, Phase 1-2 trialErratum in. Lancet Oncol (2018) 1919(23):257e137–66. 10.1016/S1470-2045(17)30912-9 PMC9098158 29276022

[11] Cole PD McCarten KM Pei Q Spira M Metzger ML Drachtman RA Brentuximab Vedotin with Gemcitabine for Paediatric and Young Adult Patients with Relapsed or Refractory Hodgkin's Lymphoma (AHOD1221): A Children's Oncology Group, Multicentre Single-Arm, Phase 1–2 Trial. Lancet Oncol (2018) 19(9):1229–38. 10.1016/S1470-2045(18)30426-1 30122620 PMC6487196

[12] Locatelli F Mauz-Koerholz C Neville K Llort A Beishuizen A Daw S Brentuximab Vedotin for Paediatric Relapsed or Refractory Hodgkin's Lymphoma and Anaplastic Large-Cell Lymphoma: a Multicentre, Open-Label, Phase 1/2 Study. Lancet Haematol (2018) 5:e450–61. 10.1016/S2352-3026(18)30153-4 30290902

[13] Maradana S Akella P Nalluru SS Jindal V Siddiqui AD. Hypertriglyceridemia Induced Pancreatitis Due to Brentuximab Therapy: First Case Report. Cureus (2019) 11(7):e5138. 10.7759/cureus.5138 31523567 PMC6741392

[14] Urru SAM Mariotti E Carta P Massidda S Marcias M Murru R Acute Pancreatitis Following Brentuximab Vedotin Therapy for Refractory Hodgkin Lymphoma: A Case Report. Drugs R D (2014) 14:9–11. 10.1007/s40268-014-0036-x 24493291 PMC3964296

[15] Gandhi MD Evens AM Fenske TS Hamlin P Coiffier B Engert A Pancreatitis in Patients Treated with Brentuximab Vedotin: A Previously Unrecognized Serious Adverse Event. Blood (2014) 123(18):2895–7. 10.1182/blood-2014-03-561878 24786458 PMC4007615

[16] Vinti L Locatelli F Merli P Parasole R Buffardi S Pillon M Brentuximab Vedotin in Combination with Bendamustine in Relapsed or Refractory Hodgkin Lymphoma: A Retrospective Analysis on 23 Paediatric Patients or Young Adults. Blood (2017) 130:1. 10.1182/blood.V130.Suppl_1.4090.4090 28684444

[17] McMillan A O'Neil AT Townsend W Lambert J Virchi A Shah R The Addition of Bendamustine to Brentuximab Vedotin Leads to Improved Rates of Complete Metabolic Remission in Children, Adolescents and Young Adults with Relapsed and Refractory Classical Hodgkin Lymphoma: A Retrospective Single-centre Series. Br J Haematol (2021) 192:e84–e87. 10.1111/bjh.17274 33426648

[18] Körholz D Wallace WH Landman-Parker J. Euro-net-Paediatric Hodgkin’s Lymphoma Group (Euro-Net-PHL-C2): Second International Inter-group Study for Classical Hodgkin Lymphoma in Children and Adolescents (2015). Available at: https://clinicaltrials.gov/ct2/show/NCT02684708 (Accessed February 20, 2022).

[19] Suri A Mould DR Song G Kinley J Venkatakrishnan K. Population Pharmacokinetics of Brentuximab Vedotin in Adult and Pediatric Patients with Relapsed/Refractory Hematologic Malignancies: Model-Informed Hypothesis Generation for Pediatric Dosing Regimens. J Clin Pharmacol (2020) 60(12):1585–97. 10.1002/jcph.1682 32596842 PMC7689911

[20] Pang Y Kartsonaki C Turnbull I Guo Y Yang L Bian Z Metabolic and Lifestyle Risk Factors for Acute Pancreatitis in Chinese Adults: A Prospective Cohort Study of 0.5 Million People. Plos Med (2018) 15(8):e1002618. 10.1371/journal.pmed.1002618 30067849 PMC6070164

[21] Aune D Mahamat-Saleh Y Norat T Riboli E. High Body Mass Index and Central Adiposity Is Associated with Increased Risk of Acute Pancreatitis: A Meta-Analysis. Dig Dis Sci (2021) 66(4):1249–67. 10.1007/s10620-020-06275-6 32556971 PMC7990844

[22] Li X Guo X Ji H Niu J Gao P. Relationships between Metabolic Comorbidities and Occurrence, Severity, and Outcomes in Patients with Acute Pancreatitis: A Narrative Review. Biomed Res Int (2019) 2019:2645926. 10.1155/2019/2645926 31687382 PMC6800961

[23] Wang SQ Shu-jun L Quan-xin F Xiang-ying F Xu L Zhao QC Overweight Is an Additional Prognostic Factor in Acute Pancreatitis: A Meta-Analysis. Pancreatology (2011) 11(2):92–8. 10.1159/000327688 21577040

[24] Evens AM Advani RH Helenowski IB Fanale M Smith SM Jovanovic BD Multicenter Phase II Study of Sequential Brentuximab Vedotin and Doxorubicin, Vinblastine, and Dacarbazine Chemotherapy for Older Patients with Untreated Classical Hodgkin Lymphoma. J Clin Oncol (2018) 36(30):3015–22. 10.1200/JCO.2018.79.0139 30179569

[25] Ching Chen C Su-Peng Y. Fatal Pancreatitis Occurred in a Patient with Refractory CD30+ Anaplastic Large Cell Lymphoma after Brentuximab Vedotin Treatment. J Cancer Res Pract (2017) 4(1):35–7. 10.1016/j.jcrpr.2016.09.002

[26] Anderson K Shehata M Singh D Al-Ourani M. Acute Pancreatitis Induced by Polatuzumab-Vedotin-Piiq in Combination with Bendamustine and Rituximab for Diffuse Large B-Cell Lymphoma. Cureus (2020) 12(9):e10299. 10.7759/cureus.10299 33047089 PMC7540405

[27] Whitcomb DC. Genetic Risk Factors for Pancreatic Disorders. Gastroenterology (2013) 144(6):1292–302. 10.1053/j.gastro.2013.01.069 23622139 PMC3684061

[28] Tenner S. Drug-Induced Acute Pancreatitis: Underdiagnosis and Overdiagnosis. Dig Dis Sci (2010) 55:2706–8. 10.1007/s10620-010-1367-2 20686844

[29] Stefanović M Jazbec J Lindgren F Bulajić M Löhr M. Acute Pancreatitis as a Complication of Childhood Cancer Treatment.. Cancer Med (2016) 5(5):827–36. 10.1002/cam4.649 26872431 PMC4864812

[30] Raja RA Schmiegelow K Frandsen TL. Asparaginase-associated Pancreatitis in Children. Br J Haematol (2012) 159:18–27. 10.1111/bjh.12016 22909259

[31] Hijiya N van der Sluis IM. Asparaginase-associated Toxicity in Children with Acute Lymphoblastic Leukemia. Leuk Lymphoma (2016) 57(4):748–57. 10.3109/10428194.2015.1101098 26457414 PMC4819847

[32] Teuffel O Kuster SP Hunger SP Conter V Hitzler J Ethier MC Dexamethasone versus Prednisone for Induction Therapy in Childhood Acute Lymphoblastic Leukemia: A Systematic Review and Meta-Analysis. Leukemia (2011) 25(8):1232–8. 10.1038/leu.2011.84 21527934

[33] Kearney SL DahlbergLevy SEDE Voss SD Sallan SE Silverman LB. Clinical Course and Outcome in Children with Acute Lymphoblastic Leukemia and Asparaginase-Associated Pancreatitis. Pediatr Blood Cancer (2009) 53:162–7. 10.1002/pbc.22076 19405141 PMC2721691

[34] Junquera Alonso E Seoane Blanco L Cano Calderero FX. Bortezomib-induced Acute Pancreatitis, an Uncommon Adverse Event. Rev Esp Enferm Dig (2021) 113(1):77. 10.17235/reed.2020.7120/2020 33207901

[35] Brulc E Seehaus C Schutz N Fantl D. Management of Pancreatitis Related to Bortezomib Treatment: Report of Two Cases. Hematol Transfus Cel Ther (2018) 40(4):382–4. 10.1016/j.htct.2018.03.003 PMC6200673 30370418

[36] Halalsheh H Bazzeh F Alkayed K Salami K Madanat F. 6-Mercaptopurine-induced Recurrent Acute Pancreatitis in Children with Acute Lymphoblastic Leukemia/Lymphoma. J Pediatr Hematol Oncol (2013) 35(6):470–2. 10.1097/mph.0b013e318271c92f 23138114

[37] Zerra P Bergsagel J Keller FG Glen L Pauly M. Maintenance Treatment with Low-Dose Mercaptopurine in Combination with Allopurinol in Children with Acute Lymphoblastic Leukemia and Mercaptopurine-Induced Pancreatitis. Pediatr Blood Cancer (2016) 63(4):712–5. 10.1002/pbc.25841 26878433

[38] McGrail LH Sehn LH Weiss RB Robson MR Antin JH Byrd JC Pancreatitis during Therapy of Acute Myeloid Leukemia: Cytarabine Related? Ann Oncol (1999) 10(11):1373–6. 10.1023/a:1008342320532 10631468

[39] Sastry J Young S Shaw P. Acute Pancreatitis Due to Tacrolimus in a Case of Allogeneic Bone Marrow Transplantation. Bone Marrow Transpl (2004) 33:867–8. 10.1038/sj.bmt.1704429 14743193

[40] Xu J Xu L Wei X Li X Cai M. A Case Report: Acute Pancreatitis Associated with Tacrolimus in Kidney Transplantation. BMC Nephrol (2019) 20:209. 10.1186/s12882-019-1395-x 31174507 PMC6555724

[41] Stock W Douer D DeAngelo DJ Arellano M Advani A Damon L Prevention and Management of Asparaginase/pegasparaginase-Associated Toxicities in Adults and Older Adolescents: Recommendations of an Expert Panel. Leuk Lymphoma (2011) 52(12):2237–53. 10.3109/10428194.2011.596963 21827361

[42] Abu-El-Haija M Kumar S Quiros JA Balakrishnan K Barth B Bitton S Management of Acute Pancreatitis in the Pediatric Population: A Clinical Report From the North American Society for Pediatric Gastroenterology, Hepatology and Nutrition Pancreas Committee. J Pediatr Gastroenterol Nutr (2018) 66(1):159–76. 10.1097/MPG.0000000000001715 29280782 PMC5755713

[43] Isenmann R Rünzi M Kron M Kahl S Kraus D Jung N Prophylactic Antibiotic Treatment in Patients with Predicted Severe Acute Pancreatitis: A Placebo-Controlled, Double-Blind Trial. Gastroenterology (2004) 126(4):997–1004. 10.1053/j.gastro.2003.12.050 15057739

[44] García-Barrasa A Borobia FG Pallares R Jorba R Poves I Busquets J A Double-Blind, Placebo-Controlled Trial of Ciprofloxacin Prophylaxis in Patients with Acute Necrotizing Pancreatitis. J Gastrointest Surg (2009) 13(4):768–74. 10.1007/s11605-008-0773-7 19082671

[45] Dambrauskas Z Gulbinas A Pundzius J Barauskas G. Meta-analysis of Prophylactic Parenteral Antibiotic Use in Acute Necrotizing Pancreatitis. Medicina (Kaunas) (2007) 43(4):291. 10.3390/medicina43040036 17485956

[46] Thu Huynh V Bergeron S. Asparaginase Toxicities: Identification and Management in Patients with Acute Lymphoblastic Leukemia. Clin J Oncol Nurs (2017) 21(5):E248–E259. 10.1188/17.CJON.E248-E259 28945721

[47] Burke PW Hoelzer D Park JH Schmiegelow K Douer D. Managing Toxicities with Asparaginase Based Therapies in Adult ALL: Summary of an ESMO Open– Cancer Horizons Roundtable Discussion. ESMO Open (2020) 5:e000858. 10.1136/esmoopen-2020-000858 33037033 PMC7549445

[48] Oak E Bartlett NL. A Safety Evaluation of Brentuximab Vedotin for the Treatment of Hodgkin Lymphoma. Expert Opin Drug Saf (2016) 15:875–82. 10.1080/14740338.2016.1179277 27139729

[49] Carson KR Newsome SD Kim EJ Wagner-Johnston ND von Geldern G Moskowitz CH Progressive Multifocal Leukoencephalopathy Associated with Brentuximab Vedotin Therapy: A Report of 5 Cases from the Southern Network on Adverse Reactions (SONAR) Project. Cancer (2014) 120:2464–71. 10.1002/cncr.28712 24771533 PMC4460831

[50] Berger JR Aksamit AJ Clifford DB Davis L Koralnik IJ Sejvar JJ PML Diagnostic Criteria: Consensus Statement from the AAN Neuroinfectious Disease Section. Neurology (2013) 80(15):1430–8. 10.1212/WNL.0b013e31828c2fa1 23568998 PMC3662270

[51] EMA Europa. Adcetris. [Internet] (2022). Available from: https://www.ema.europa.eu/en/documents/product-information/adcetris-epar-product-information_en.pdf (Accessed February 23, 2022).

